# Detection of mutations in circulating cell‐free DNA in relation to disease stage in colorectal cancer

**DOI:** 10.1002/cam4.2219

**Published:** 2019-05-27

**Authors:** Sandra Liebs, Ulrich Keilholz, Inge Kehler, Caroline Schweiger, Johannes Haybäck, Anika Nonnenmacher

**Affiliations:** ^1^ German Cancer Consortium (DKTK) German Cancer Research Center (DKFZ) Heidelberg Germany; ^2^ Charité ‐ Universitätsmedizin Berlin, corporate member of Freie Universität Berlin Humboldt-Universität zu Berlin, and Berlin Institute of Health Charité Comprehensive Cancer Center Berlin Germany; ^3^ Institute of Pathology, Medical University Graz Graz Austria

**Keywords:** *BRAF*, circulating cell‐free DNA, colorectal cancer, *KRAS*

## Abstract

Enthusiasm has emerged for the potential of liquid biopsies to provide easily accessible genetic biomarkers for early diagnosis and mutational cancer characterization. We here systematically investigated the suitability of circulating cell‐free DNA (cfDNA) analysis for mutation detection in colorectal cancer (CRC) patients with respect to clinicopathological disease stage. Droplet Digital PCR (ddPCR) was performed to detect common point mutations in the *KRAS* and *BRAF* oncogenes in cfDNA from 65 patients and compared to mutations in tumor tissue. Stage of disease was classified according to UICC (Union for International Cancer Control) criteria. In tumor tissue, *KRAS* or *BRAF* mutations were present in 35 of 65 cases (44% UICC stage I, 50% stage II, 47% stage III, and 62% stage IV). Although cfDNA was detected in 100% of patients, ddPCR displayed the tumor tissue mutation in only 1 of 6 (17%) stage II patients, whereas 10 of 18 (56%) reported variants were verified in cfDNA samples of the stage IV cohort. No *BRAF* or *KRAS* mutation was detected in cfDNA from patients with wild‐type tumor tissue. In one case of mutant stage II colon cancer (*KRAS*‐G12C), the G12D variant was detected in cfDNA instead. Further workup revealed that circulating tumor‐derived DNA and liver metastases originated from a synchronous *KRAS*‐mutated cancer of the pancreas. Our results demonstrate that ddPCR‐based analysis is highly specific and useful for mutation monitoring, but the sensitivity limits its usefulness for early cancer detection.

## 1 INTRODUCTION

Colorectal cancer (CRC) is the second most common cause of cancer death in Europe.^1^ The 5‐year survival rate of 92% in stage I cancer patients decreases to 12% in those present with distant metastasis, demonstrating the crucial need for early detection and treatment.^2^ Up to 40% of CRC patients are unlikely to benefit from EGFR‐targeted therapies, such as cetuximab and panitumumab, due to mutations in the *KRAS* oncogene.^3^ Even early responders with *RAS* wild‐type tumors develop secondary resistance under pressure of EGFR‐directed treatments due to emerging tumor subclones.^4^ Furthermore, 8%‐15% of CRC patients with *KRAS* wild‐type tumors harbor *BRAF* mutations, which have been proven to be an additional negative predictor of response to anti‐EGFR treatment. Given that, patient management requires mutational monitoring of the disease as a basis for personalized medicine. In clinical practice, tissue biopsies are obtained for molecular profiling although a fragment of a single lesion might be inadequate to reflect intratumoral heterogeneity presented at low frequencies. Therefore, blood‐based mutational profiling is suggested as a promising approach to provide a more comprehensive molecular profile of the disease in a minimally invasive manner. Liquid biopsy includes the analysis of tumor‐derived biomarkers in any body fluid, such as plasma, urine, and cerebrospinal fluid. In particular, serial blood testing is proposed as a convenient real‐time tool to identify spatial and temporal heterogeneity predicting response or resistance to targeted agents.^5^

Circulating cfDNA is composed of small nucleic acid fragments liberated from cells by rupture, necrosis or apoptosis originating from normal and deceased cells. Thus, circulating tumor‐derived DNA (ctDNA) is only identified via the detection of cancer‐related mutations. In correlation with tumor burden, mutant allele frequencies were reported to range between less than 10 and up to 1000 mutant copies per 5 mL plasma in stage I‐IV cancer patients,^6^ suggesting limitations in early stage cancer. We here systematically investigated the sensitivity and specificity of the analysis of somatic mutations in plasma samples from CRC patients in relation to disease stage. Since circulating tumor cells (CTC) can provide an alternative source of genetic information in liquid biopsies, the mutation detection in cfDNA was compared with the presence of CTCs.

## 2 MATERIAL AND METHODS

### 2.1 Patients

Patients with early and advanced CRC were included in the OncoTrack research project at the Charité and the Medical University Graz between 2010 and 2016.^7^ Informed consent was obtained prior to blood and tissue specimen collection. The study was approved by the ethics committee of the Charité University Medicine (Charitéplatz 1, 10117 Berlin, Germany; EA 1/069/11). It was also approved and confirmed by the ethics commission of the Medical University of Graz (Auenbruggerplatz 2, 8036 Graz, Austria) and the ethics committee of the St John of God Hospital Graz (23‐015 ex 10/11), respectively. Disease stage was classified according to the criteria of the Union for International Cancer Control (7th edition).^8^

### 2.2 Cell lines

DNA isolated from human‐derived cell lines with reported wild‐type or mutation status in the oncogenes *KRAS* and *BRAF* was used to establish Droplet Digital PCR (ddPCR) assays (Table S1). All cell lines were cultured in media supplemented with 10% heat‐inactivated fetal bovine serum (Thermo Fisher Scientific, Waltham, USA) and 1% penicillin/streptomycin (Biochrom GmbH, Berlin, Germany) at 37°C and 5% CO_2_. Prior to DNA isolation, cell lines were tested negative for mycoplasma using the Promokine PCR Mycoplasma Test KIT I/C following manufacturer's specifications (PromoCell GmbH, Heidelberg, Germany). Cell line authenticity was validated by single nucleotide polymorphism profiling with Multiplexion GmbH (Friedrichshafen, Germany). Following manufacturer's instructions, the GeneJET Genomic DNA Purification kit (Thermo Scientific, Waltham, USA) was used to isolate DNA eluted in 100 µL double distilled water.

### 2.3 Nucleic acid preparation from blood and tissue specimens

Prior to tumor resection, peripheral blood samples were collected in BD Vacutainer® PST™ II heparin tubes (BD, Franklin Lakes, USA) and directly processed by centrifugation for initial plasma storage at −80°C (1500 *g* for 10 minutes or 10 minutes at 800 *g* followed by 1600 *g* for 10 minutes). Furthermore, heparin blood from six healthy donors was centrifuged at 1811 *g* for 7 minutes followed by 3061 *g* for 10 minutes. In 2010, when patient recruitment started for the OncoTrack research project, the knowledge about stabilizing ctDNA in plasma samples was not as advanced as it is today. Most publications regarding the superior effect of EDTA and other blood collection tubes on preserving cfDNA and CTCs, while preventing hematopoietic cells from lysis, were published since 2016.^9^, ^10^ In 2004, Lam et al reported that EDTA is a superior anticoagulant compared to heparin, but only when blood processing was delayed, whereas comparable results regarding DNA concentrations were obtained when plasma was isolated within 6 hours after blood draw.^11^ In our study, plasma was directly isolated after blood collection. Furthermore, at the time of patient recruitment, internal analysis in our group demonstrated comparable DNA concentrations when using EDTA and heparin collection tubes, which, however, was not published. Based on this knowledge, we decided to use the stored plasma samples from the OncoTrack project for the analysis of cfDNA. A cfDNA assay system developed for heparin blood samples was employed. All plasma samples were centrifuged at 2000 *g* for 15 minutes prior to cfDNA isolation using the QIAamp DNA Blood Midi kit (Qiagen, Hilden, Germany). Briefly, 0.4‐3.0 mL plasma was incubated with protease and Buffer AL for 10 minutes at 70°C, transferred to the QIAamp Midi column and washed with Buffer AW1 and AW2 at 4258 g for 1 minute and 15 minutes, respectively. Nucleic acid was eluted in 250 µL Ultra Pure water and further concentrated to 55 µL using Zymo's DNA Clean & Concentrator®‐5 kit according to the protocol specifications (Irvine, USA).

Within the large scale deep sequencing program of OncoTrack, whole genome and whole exome sequencing of tumor tissue specimens was performed, resulting in an accessible database of omics data.^7^ In tissue samples not sequenced within the OncoTrack program, variant detection was performed using the same ddPCR assay as for cfDNA samples. Ten micrometer thick formalin‐fixed paraffin‐embedded (FFPE) tissue slides were deparaffinized and processed following the specifications of the High Pure FFPET DNA Isolation kit (Roche, Basel, Switzerland). The GeneJET Genomic DNA Purification kit (Thermo Scientific, Waltham, USA) was used to isolate DNA from fresh frozen tissue following the manufacturer's instructions using the double amount of enzymatic solutions. Digestion of tumor tissue was performed for 2 hours at 56°C each. After purification, DNA from fresh frozen tissue was eluted in 150 µL double distilled water, whereas FFPE‐derived DNA was eluted in 30 µL.

### 2.4 DNA quantification and fragment analysis

DNA concentrations were quantified using the DeNovix DS‐11 FX+ (Biozym Scientific GmbH, Hessisch Oldendorf, Germany). DNA isolated from fresh frozen tissue and cell lines was quantified via UV‐Vis absorbance, whereas concentrations of FFPE‐derived and circulating cell‐free DNA (cfDNA) were determined using the Qubit® dsDNA HS Assay kit (Thermo Fisher Scientific). Additionally, fragment length of cfDNA was analyzed on the Agilent 2100 Bioanalyzer using the High Sensitivity DNA Kit (Agilent, Eugen, USA). To exclude cfDNA from normal cells of higher fragment size from tumor‐derived DNA fragments, the correlation area under the curve in the region from 50‐260 bp was determined to compare the resulting cfDNA concentrations (ng/mL) between patients of different tumor stages.

### 2.5 Variant detection via ddPCR

Expecting low allele frequencies of mutant variants in cfDNA, the highly sensitive Droplet Digital™ PCR platform was used for mutation detection (Bio‐Rad Laboratories GmbH, Munich, Germany). Our study design consisted of two parts: a first evaluation of 2‐3 somatic mutations or the wild‐type of the *KRAS* oncogene detected via a multiplex assay and a verification duplex PCR only detecting the wild‐type or one of the mutations. Two *KRAS* multiplex assays were designed, one detected variants G12D/A or G13D (*KRAS* I multiplex) whereas the second assay detected G12V/C (*KRAS* II multiplex). Differentiation between mutations in multiplex assays was enabled by using different concentrations of FAM‐labeled probes whereas the wild‐type was detected with a HEX‐labeled probe. Due to our main focus on the V600E variant of the *BRAF* gene, only a duplex PCR was used here for sample testing without further verification. Primers and probes were designed and tested for specificity using the Primer3, Primer‐BLAST, and UCSC In‐Silico PCR software.^12^, ^13^, ^14^

Each ddPCR reaction mixture was prepared using 3 µL DNA and 17 µL mastermix containing 2X ddPCR Supermix for Probes with no dUTP (Bio‐Rad Laboratories GmbH, Munich, Germany), each primer at final concentrations of 900 nM and probe concentrations as listed in Table S2. Analyzing cell line‐derived gDNA as control samples, EcoRI‐HF (New England Biolabs) was further added to the reaction mix resulting in a final enzyme concentration of 0,5 units/µL. Droplets were generated using the QX200 Droplet generator, manually transferred to a 96‐well PCR plate (Eppendorf, Hamburg, Germany) and heat‐sealed with the PX1 Plate Sealer (Bio‐Rad). PCR reactions were performed in the T‐100 thermal cycler (Bio‐Rad) with the following program: 1 cycle at 95°C for 10 minutes, 40 cycles at 94°C for 30 seconds and at 56°C or 59°C for 1 minute (*BRAF* or *KRAS* assays, respectively), and 1 cycle at 98°C for 10 minutes. Droplets were read in the QX200 Droplet Reader (Bio‐Rad) and analyzed using the QuantaSoft software (version 1.7.4, Bio‐Rad). Patient‐derived samples were analyzed in duplicates. Each run included nontemplate controls to exclude the presence of contaminations. Cell line‐derived gDNA harboring the mutations of interest were diluted in wild‐type gDNA with a frequency of 1% to demonstrate successful target amplification within each run.

### 2.6 Determination of assay performance and evaluation strategy of ddPCR results

False‐positive rate (FPR) and limit of detection (LOD) were determined for multiplex and duplex assays. FPR was evaluated by determining the number of unspecific events in the mutation channel when analyzing nontemplate controls and only wild‐type cell line‐derived DNA samples with many and few copies per microliter adjusted to expectant cfDNA levels (500 and 100 cpm, respectively). All assays demonstrated a FPR of 0 to 0.8 events, resulting in a defined cutoff value of one event. Mutant gDNA was diluted in constant wild‐type gDNA (ranging from 10% to 0.001%), identifying a LOD of 0.01% for all established assays.

The evaluation strategy is depicted in Figure S1A. Briefly, only samples with ≥10.000 generated droplets were included into the final analysis. Two dimensional plots of gDNA samples derived from cell lines harboring the mutation of interest were used for first threshold setting, which was corrected if necessary, using the 1D plot. Outliers regarding high‐fluorescence signals were excluded during quantification of positive events. Events in the wild‐type and mutation channel were quantified and evaluated by being dispersed or overlapping with the positive controls in the 2D plot. Despite an FPR of one event in the multiplex set up, when analyzing the complete data set, three or more events in the multiplex PCR were proven to be positive in the validation duplex as well.

### 2.7 Circulating tumor cell enrichment and quantification

Up to 50 mL of whole blood was collected in BD Vacutainer® heparin tubes for the enrichment and detection of circulating tumor cells. Between 8 and10 mL of whole blood was added to 40 mL of 1X Red blood cell lysis buffer (Stemcell Technologies, Vancouver, Canada) and incubated at room temperature for a maximum of 15 minutes. Remaining cells were washed with PBS (290 *g*, 5 minutes, 4°C) and resuspended in PBS containing 2% FCS and 2 mM EDTA to a concentration of ≤5 × 10^7^ cells per milliliter for subsequent CD45 depletion using the EasySep™ Human CD45 Depletion kit (Stemcell Technologies). Incubation with the CD45‐recognizing tetrameric antibody complex as well as the incubation with the magnetic particles was performed at 4°C for 15 minutes each. Labeled cells were separated using the EasySep™ magnet for 5 minutes at room temperature. The depleted cell fraction was washed and resuspended in 100 µL PBS prior to incubation with 10 µL FcR blocking reagent (Miltenyi Biotec, Bergisch Gladbach, Germany) for 10 minutes at 4°C. To discriminate remaining leukocytes from tumor cells, an antibody against IgG1‐AF555 (1 µL Life Technologies, Carlsbad, USA) recognizing the CD45 depletion cocktail as well as anti‐EpCAM‐AF488 (2 µL Biolegend, San Diego, USA) and anti‐CEA‐AF488 (2 µL Biolegend) were incubated for 20 minutes at 4°C. Additionally, 2 µL LIVE/DEAD™ Fixable Blue Dead Cell Stain for UV excitation (Life Technologies) was incubated for 10 minutes at 4°C to identify dead cells. Tumor cell quantification was performed using the DMI3000B fluorescence microscope (Leica, Wetzlar, Germany), whereby only living cells positive for EpCAM and/or CEA but negative for CD45 were identified as CTCs.

### 2.8 Statistical analysis

Categorical variables were summarized by frequency and continuous variables by median and range. Assay performance was evaluated by the detection of reported *KRAS* and *BRAF* tissue mutations in cfDNA samples (sensitivity) and by confirming plasma samples determined as wild‐type from the tissue analysis (specificity).

## 3 RESULTS

### 3.1 Characteristics of the patient cohort

From the OncoTrack cohort, 65 plasma samples collected prior to treatment and resection of tissue specimens were accessible for cfDNA isolation. Patients' median age was 67 years (range 36‐92 years). Thirty‐nine patients were male (60%) and 26 were female (40%). Ten patients (15%) had tumors with a *BRAF* V600E mutation and 25 patients (38%) had tumors with *KRAS* mutations in codon 12 or 13 (G12D/V/C or G13D). Patients with a reported *BRAF* mutation were presumed to harbor *KRAS* wild‐type and vice versa, since coexistence of mutations in both oncogenes occurs with a probability of only 0.0001%.^15^ A detailed overview of patients' clinicopathological characteristics was presented in Table 1.

**Table 1:** Demographic and clinical characteristics of study participants

Characteristics	Total	Stage I	Stage II	Stage III	Stage IV
Number of patients	N = 65	N = 9	N = 12	N = 15	N = 29
Age at enrollment, years					
Median	67	67	69	70	63
Range	36‐92	49‐79	46‐79	39‐83	36‐92
Sex, n (%)					
Male	39 (60%)	6 (67%)	7 (58%)	10 (67%)	16 (55%)
Female	26 (40%)	3 (33%)	5 (42%)	5 (33%)	13 (45%)
Tissue gene status, n (%)					
*KRAS*‐MUT	25 (38%)	2 (22%)	2 (17%)	6 (40%)	15 (52%)
*BRAF*‐MUT	10 (15%)	2 (22%)	4 (33%)	1 (7%)	3 (10%)
WT	18 (28%)	2 (22%)	4 (33%)	5 (33%)	7 (24%)
Unknown	12 (18%)	3 (33%)	2 (17%)	3 (20%)	4 (14%)
CTC detection rate, n (%)					
Performed CTC analysis	54 (83%)	7 (78%)	12 (100%)	13 (87%)	22 (76%)
Patients with CTCs	29 (54%)	4 (57%)	7 (58%)	8 (62%)	10 (45%)
Patients without CTCs	25 (46%)	3 (43%)	5 (42%)	5 (38%)	12 (55%)
CTC numbers					
Median	1	1	2	1	0
Range	0‐8	0‐4	0‐8	0‐6	0‐5
Not available	11	2	0	2	7

*KRAS*‐MUT includes the G12D, G12V, G12C and G13D variants, whereas *BRAF*‐MUT refers to the V600E mutation.

Abbreviation: CTC, circulating tumor cells.

### 3.2 Quantitative analysis of cfDNA

**Figure 1:**
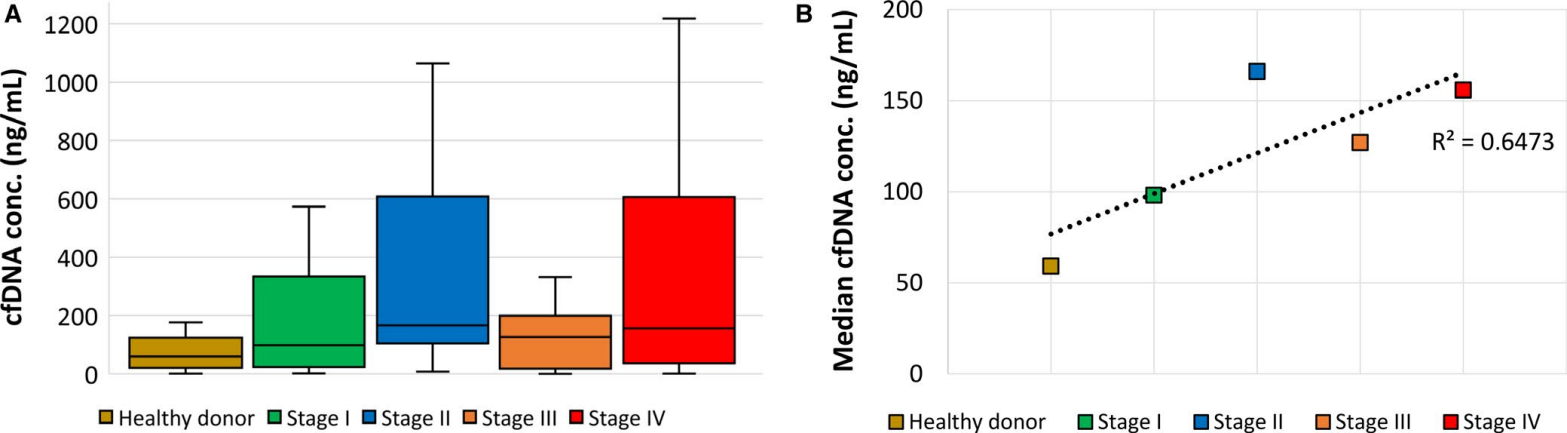
Cell‐free DNA (cfDNA) concentrations und mutation detection in relation to disease stage. A, Quantitative analysis of cfDNA isolated from stage I‐IV colon cancer patients in comparison to healthy individuals. Box plot showing median, first and third quartiles with whiskers from minimum to maximum. B, Median cfDNA levels demonstrate an increase with higher tumor burden

**Figure 2:**
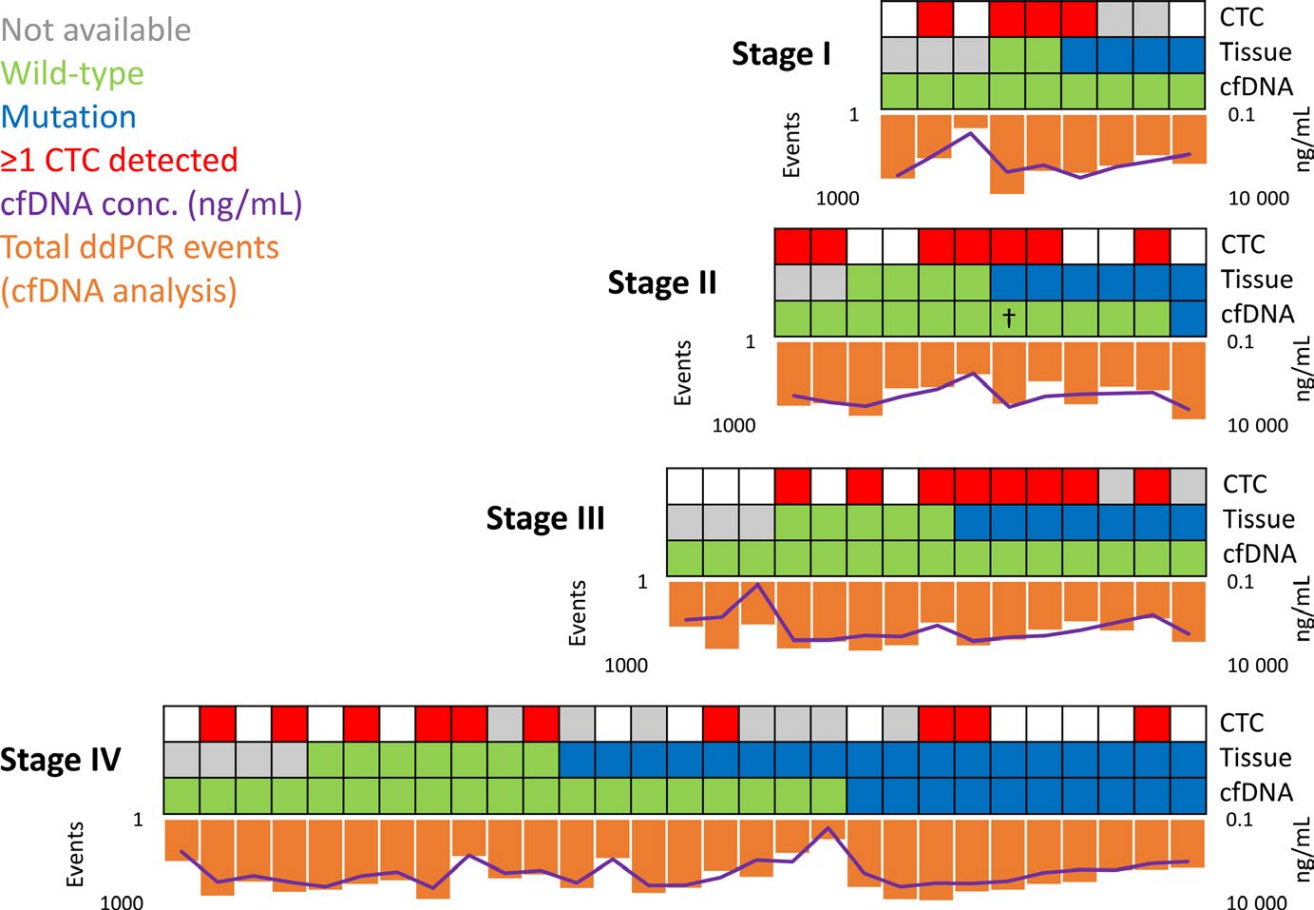
Retrieval of tissue‐reported mutations in plasma in comparison to cell‐free DNA (cfDNA) concentration and the detection of circulating tumor cells (CTCs). Corresponding to higher cfDNA levels, total Droplet Digital PCR detection events in the wild‐type and mutation channel increased, which, however, did not correlate with successful retrieval of tissue‐reported variants in plasma. CTCs were detected in blood samples from patients of all cancer stages, highlighting that the analysis of tumor‐derived cells in the periphery will possibly complement the limited information received by cfDNA analysis. ^†^The CRC‐derived *KRAS* mutation (G12C) was not verified in plasma from patient 374‐CB‐M; however, the G12D variant originating from the synchronous stage IV cancer of the pancreas was detected

Quantitative analysis of cfDNA samples demonstrated an increase in cfDNA concentrations with higher tumor burden varying from 59 ng/mL in healthy individuals to 156 ng/mL in patients with metastasized colon cancer (Figure 1A, 1). Correlating with increasing cfDNA level, ddPCR analysis detecting the *BRAF* and *KRAS* oncogenes resulted in higher events in the wild‐type and mutation channel, which, however, did not correlate with successful tissue mutation retrieval in cfDNA samples (Figure 2). Highly concentrated cfDNA samples did not necessarily present circulating tumor DNA.

### 3.3 Mutation status analysis from tumor tissue and plasma

Within our study cohort, *KRAS* or *BRAF* mutations were present in 35 of 65 (54%) tumor specimens. cfDNA was detected in 100% of patients independently of plasma volume or DNA concentration. No correlation between plasma volume and successful ctDNA detection was observed. Comparably low plasma volumes (≤0.5 mL) were available from only three patients harboring a tissue mutation; however, the cfDNA concentration from only one of them was very limited (22.86 ng/mL) possibly explaining the absence of the tumor‐derived mutation in plasma (Table S3). Mutational profiling of cfDNA verified CRC‐related mutations in 11 of 35 (31%) corresponding plasma samples (Figure 2), including 2 of 10 (20%) *BRAF* and 9 of 25 (36%) *KRAS* mutations. Independently of tumor stage, mutant allele frequencies ranged between 0.01 and 0.52 (more than 50‐fold) with mutations detected with 2 to 227 ddPCR events (more than 100‐fold). Individual data for each mutant cfDNA sample are shown in Figure S1B. No *BRAF* or *KRAS* mutation was detected in cfDNA from patients with wild‐type tumor tissue. Thus, ddPCR assays showed 100% specificity throughout all stages with increasing accuracy in patients with higher tumor burden (Table 2). However, sensitivity was very limited with a maximum of 56% in stage IV patients. Only 1 of 17 (6%) CRC‐derived gene variants was verified in all stage I‐III patients. In stage II patient 249‐CB‐P, the *BRAF* mutation in the tumor was detected in the corresponding plasma sample with an allele frequency of 0.05 (27 mutation events). In comparison to cfDNA levels of the remaining stage II patients (median: 143.6 ng/mL), 249‐CB‐P demonstrated a strikingly higher concentration (1064.25 ng/mL), increasing the possibility of successful ctDNA detection.

**Table 2:** *BRAF* and *KRAS* gene status concordance between tumor tissue and cfDNA

	Total (N = 53)		cfDNA analysis							
			Stage I		Stage II		Stage III		Stage IV	
	MUT	WT	MUT	WT	MUT	WT	MUT	WT	MUT	WT
Tissue analysis										
MUT	11	24	0	4	1	5	0	7	10	8
WT	0	18	0	2	0	4	0	5	0	7
Sensitivity	31%		0%		17%		0%		56%	
Specificity	100%		100%		100%		100%		100%	
Accuracy	55%		33%		50%		42%		68%	

Abbreviation: cfDNA, cell‐free DNA.

### 3.4 Discordance between colon tissue and cfDNA

There was one discrepancy in the *KRAS* gene status between the colon tumor tissue and cfDNA. Enrolled in the OncoTrack study with an adenocarcinoma of the colon and synchronous liver metastasis, the *KRAS* G12C variant detected in the primary tumor was not displayed in the corresponding cfDNA from patient 374‐CB‐M (Figure S1C, D). In contrast, the *KRAS* G12D mutation was found in plasma with an allele frequency of 0.1 (15 mutation events) as well as in the metastatic tissue. This indicated that both ctDNA and the metastasis were originated from the synchronous stage IV cancer of the pancreas, which was further verified pathologically.

### 3.5 Circulating tumor cells

Blood samples for CTC quantification were available from 54 of 65 patients (83%), the tissue status of whom was known for 42 patients. CTCs were enriched from 50 mL whole blood and identified via fluorescence microscopy detecting EpCAM and/or CEA tumor marker expression. In 29 of 54 patients (54%), CTCs were successfully detected independently from tumor stage with a range of 1‐8 CTCs per patient (Table 1). The detection of ctDNA was rather limited to patients with stage IV cancers, whereas circulating tumor‐derived cells were detected even in patients with nonmetastasized CRC (Figure 2), emphasizing the differences between cfDNA and CTCs, making them not equivalent but complementary biomarkers for prognosis of the overall cancer disease for clinical management.

## 4 DISCUSSION

One of the most desirable clinical applications of cfDNA analysis might be cancer diagnosis prior to metastatic spread, allowing early treatment to improve patients' survival. In recent years, different studies demonstrated the prognostic value of cfDNA in the breast, pancreatic, prostate, and CRC^16^, ^17^, ^18^, ^19^ further hypothesizing that its analysis might identify patients with localized tumors who are at risk of recurrence. Therefore, our study systematically investigated the utility of cfDNA to reflect molecular characteristics of the underlying disease with respect to tumor stage. Our assays have proven the highest precision with all variants detected in cfDNA being consistent with reported tissue status, except for one patient with stage II cancer of the right flexure of the colon. Here, cfDNA analysis revealed the *KRAS* mutation of the synchronous stage IV cancer of the pancreas. No *BRAF* or *KRAS* mutation was detected in cfDNA from patients with wild‐type tumor tissue, resulting in 100% assay specificity among all four cancer stages. However, we observed a considerable difference in sensitivities regarding the retrieval of known mutations from tissue in cfDNA between patients of different tumor burden. No mutations were detected in cfDNA in stage I and stage III patients and only 1 of 6 mutations was verified in the stage II cohort. Highest accuracy (68%) was achieved in patients with distant metastases, demonstrating that cfDNA analysis in patients with noninvasive cancer is limited.

Beije et al concluded that performance of ctDNA detection assays varies, inter alia, according to the methods applied. When comparing various targeted detection assays in paired samples of cfDNA and tumor tissue from 12 mCRC patients, sensitivity was highest with digital PCR.^20^ Here, 13 of 14 mutations (93%) observed in the primary tumor and/or the metastases were also detected in cfDNA. In contrast, next generation sequencing retrieved only a limited number of reported variants with a concordance between cfDNA and primary tumor and the metastasis of 39% and 55%, respectively. Guo et al used panel sequencing to detect tissue‐matched mutations in cfDNA of 56 early‐stage and advanced‐stage patients with nonsmall cell lung cancer (NSCLC). They reported an overall concordance rate of 54.6% and 80%, respectively.^21^ Of particular importance is their observation that the concordance rate can be strongly affected by multiple pre‐analytical, analytical, and biological factors. Regarding that, we might explain the sporadic mutation detection in our patient cohort with limitations, such as sample age and inconsistent processing, storing, and delivery conditions at two different hospitals. Furthermore, due to blood being collected in heparin vacutainers, we used the QIAamp DNA Blood Midi kit for cfDNA isolation. In contrast to other isolation kits, such as the QIAamp Circulating Nucleic Acid kit, the QIAamp DNA Blood Midi kit is reported to be inferior regarding the isolation of short‐fragmented ctDNA.^22^ Considering that there is room for improvement in study design, different studies confirmed that ctDNA concentrations increase with tumor size and cancer stage.^23^ This is consistent with the analysis of Bettegowda et al, who revealed a 47% sensitivity of *KRAS* mutation detection in cfDNA in stage I CRC patients, which increased to 87% in stage IV cancer.^6^ Diehl et al reported that the number of mutant *APC* gene molecules in the circulation of CRC patients depends on tumor stage being as little as 0.01% in stage I patients.^24^ Although the detection limit of our assays theoretically allowed for variant detection of an allelic frequency of 0.01%, the total amount of detected *KRAS* or *BRAF* molecules was so low in plasma samples of the stage I cohort that mutation detection would be below the FPR.

Taken together, we have confidence in the reliability when detecting a cancer‐related mutation in plasma, however, the absence of detectable mutant molecules does not eliminate the occurrence of genomic alterations in blood possibly undiscovered due to low allelic frequency or technical limitations. Those challenges highlight the urgent need of standard operating protocols to guarantee optimal sample management regarding storage, processing and analysis of plasma samples. Furthermore, most studies complement their method of choice by enlarging their panel of cancer‐related genes further including the detection of methylation patterns or circulating proteins, resulting in a more robust approach toward earlier cancer detection and disease monitoring.^25^, ^26^ In our case, we strongly recommend the use of CTCs and cfDNA as complementary biomarkers as we successfully detected circulating tumor cells in 29 of 54 patients (54%) independently of tumor burden. Inter‐ and intratumoral heterogeneity remains a challenge in cancer treatment, emphasizing the importance of individualized therapy. Therefore, liquid biopsy comprising the analysis of CTCs and cfDNA as a complementary approach holds great potential for precision cancer medicine.

## 5 CONCLUSION

In the last decade, administration of targeted therapies improved cancer patient management. Nevertheless, real‐time detection of mechanisms of early and acquired resistance is still needed, requiring accurate biomarkers that can be applied in a minimally invasive manner. The analysis of cfDNA has proven to be convenient regarding sample preservation and processing. However, its analysis for early diagnosis and monitoring of patients with localized and advanced tumor is still of limited value, even though method sensitivities and specificities are constantly improving. Mutation detection in plasma was only sporadically successful in our stage I‐III cohort, whereas only in patients with distant metastasis 68% concordance between tissue and cfDNA was demonstrated. Therefore, we hypothesize that a multi‐marker approach, such as molecular profiling of cfDNA and CTCs, might be an alternative surrogate for tissue analysis to monitor an evolving genomic landscape of tumor cells and adapt treatment regimens accordingly.

## Data Availability

The data that support the findings of this study are available from the corresponding author upon reasonable request.
