# Supplementary material for: Detection of mutations in circulating cell‐free DNA in relation to disease stage in colorectal cancer
Source: Cancer Med. 2019 May 27;8(8):3761–9. doi: 10.1002/cam4.2219 (PMC6639174; doi:10.1002/cam4.2219)
Supplement: Supplementary file 2 [file CAM4-8-3761-s002.docx]

**Supplementary Table 1: Human-derived cell lines.**

| **Cell line** | **Disease** | **Zygosity** | **Gene mutation** | **Medium** | **Source** |
| --- | --- | --- | --- | --- | --- |
| HELA | Cervix carcinoma | homozygous | Wild-type | DMEM | DSMZ |
| LS174T | Colon adenocarcinoma | heterozygous | *KRAS* G12D | EMEM | Sigma-Aldrich |
| SK-MEL-28 | Malignant melanoma | homozygous | *BRAF* V600E | RPMI-1640 | ATCC |
| HCT-116 | Colon carcinoma | heterozygous | *KRAS* G13D | RPMI-1640 | DSMZ |
| SW480 | Colon adenocarcinoma | homozygous | *KRAS* G12V | RPMI-1640 | ATCC |
| MIA PaCa-2 | Pancreas carcinoma | homozygous | *KRAS* G12C | DMEM | ATCC |
| RPMI-8226 | Multiple myeloma | heterozygous | *KRAS* G12A | RPMI-1640 | DSMZ |

DMEM: Dulbecco`s modified Eagle medium, EMEM: Eagle`s minimal essential medium, RPMI: Roswell Park Memorial Institute, DSMZ: German Collection of Microorganisms and Cell Cultures (Braunschweig, Germany), Sigma-Aldrich (Merck KGaA, Darmstadt, Germany), ATCC: American Type Culture Collection (Manassas, USA)

**Supplementary Table 2: Primer and probe sequences, final assay concentrations and annealing temperatures for Multiplex and Duplex ddPCR assays used for *KRAS* and *BRAF* variant detection**

| **Primer/Probe** | **Sequence (5’ – 3’)** | **Probe conc.** | **Annealing**  **Temp.** | **Amplicon**  **length** |
| --- | --- | --- | --- | --- |
| ***KRAS* I**  FWD  REV  WT  G12D  G13D  G12A | TTCATTATTTTTATTATAAGGCCTGC  TTAGCTGTATCGTCAAGGCA  HEX-TGTGGTAGTTGGAGCTGGTGGC-BHQ1  FAM-TGTGGTAGTTGGAGCTGATGGC-BHQ1  FAM-TGTGGTAGTTGGAGCTGGTGAC-BHQ1  FAM-TGTGGTAGTTGGAGCTGCTGGC-BHQ1 | 900nM  900nM  200nM  500nM  500nM  100nM^†^ | 59°C | 101 bp |
| ***KRAS* II**  FWD  REV  WT  G12C  G12V | TTCATTATTTTTATTATAAGGCCTGC  TTAGCTGTATCGTCAAGGCA  HEX-TGTGGTAGTTGGAGCTGGTGGC-BHQ1  FAM-TGTGGTAGTTGGAGCTTGTGGC-BHQ1  FAM-TGTGGTAGTTGGAGCTGTTGGC-BHQ1 | 900nM  900nM  200nM  500nM  250nM | 59°C | 101 bp |
| ***BRAF***  FWD  REV  WT  V600E | TTTACTTACTACACCTCAGATATA  ACTGTTCAAACTGATGGGA  HEX-TTTGGTCTAGCTACAGTGAAATCTCG-BHQ1  FAM-TTTGGTCTAGCTACAGAGAAATCTCG-BHQ1 | 900nM  900nM  200nM  500nM | 56°C | 115 bp |

†The only difference between multiplex and duplex assays is that the G12A duplex assay is performed using a final mutation probe concentration of 250nM.

**Supplementary Table 3: Individual data of each patient regarding disease stage, tissue status and corresponding liquid biopsy analysis including cfDNA and CTCs.**

| **Patient ID** | **UICC stage** | **Tissue** | | **Plasma (ml)** | **Cell-free DNA** | | | **Total CTCs** |
| --- | --- | --- | --- | --- | --- | --- | --- | --- |
|  |  | **Gene status** | **MAF** |  | **ng/ml** | **Gene status** | **MAF** |  |
| 231-CB-P | I | Unknown | - | 0.9 | 415.98 | WT | - | 0 |
| 174-CB-P | I | Unknown | - | 0.9 | 24.45 | WT | - | 4 |
| 200-CB-P | I | Unknown | - | 0.9 | 1.21 | WT | - | 0 |
| 388-CB-P | I | WT | - | 1.0 | 252.22 | WT | - | 1 |
| 253-CB-P | I | WT | - | 0.9 | 98.12 | WT | - | 1 |
| 115-CB-P | I | *BRAF* V600E | 0.09 | 0.9 | 573.48 | WT | - | 3 |
| 323-MB-P (A) | I | *KRAS* G12D | 0.40 | 1.8 | 123.63 | WT | - | NA |
| 261-MB-P (A) | I | *BRAF* V600E | 0.51 | 1.0 | 54.26 | WT | - | NA |
| 148-ML-P (A) | I | *KRAS* G12D | 0.19 | 0.8 | 22.36 | WT | - | 0 |
| P151 BP | II | Unknown | - | 1.0 | 157.13 | WT | - | 8 |
| 281-CB-P | II | Unknown | - | 0.9 | 397.68 | WT | - | 2 |
| 266-CB-P | II | WT | - | 0.9 | 678.55 | WT | - | 0 |
| 246-CB-P | II | WT | - | 0.9 | 180.50 | WT | - | 0 |
| 377-CB-P | II | WT | - | 0.8 | 65.21 | WT | - | 1 |
| 375-CB-P | II | WT | - | 0.8 | 7.60 | WT | - | 3 |
| 374-CB-M† | II | *KRAS* G12C | 0.19 | 2.0 | 779.07 | WT | - | 4 |
| 181-MB-P (A) | II | *BRAF* V600E | 0.29 | 0.5 | 175.02 | WT | - | 5 |
| 238-CB-P | II | *KRAS* G12D | 0.63 | 0.8 | 130.07 | WT | - | 0 |
| 265-CB-P | II | *BRAF* V600E | 0.34 | 0.9 | 112.74 | WT | - | 0 |
| 157-MB-P (A) | II | *BRAF* V600E | 0.24 | 0.9 | 102.24 | WT | - | 3 |
| **249-CB-P** | **II** | ***BRAF* V600E** | **0.08** | **0.8** | **1064.25** | ***BRAF* V600E** | **0.05** | **0** |
| 205-CB-M | III | Unknown | - | 0.9 | 17.64 | WT | - | 0 |
| 258-CB-M | III | Unknown | - | 0.8 | 12.16 | WT | - | 0 |
| 381-CB-P | III | Unknown | - | 0.9 | 0.14 | WT | - | 0 |
| 393-CB-P | III | WT | - | 0.9 | 295.23 | WT | - | 1 |
| P146 BP | III | WT | - | 0.9 | 289.67 | WT | - | 0 |
| 380-CB-P | III | WT | - | 1.0 | 159.69 | WT | - | 3 |
| 399-CB-P | III | WT | - | 1.1 | 181.27 | WT | - | 0 |
| 396-CB-P | III | WT | - | 1.2 | 39.17 | WT | - | 1 |
| 118-CB-P | III | *KRAS* G12D  *KRAS* G13D | 0.44  0.87 | 0.9 | 331.86 | WT | - | 2 |
| 280-CB-P | III | *KRAS* G13D | 0.43 | 0.9 | 199.53 | WT | - | 4 |
| 155-MB-P (A) | III | *KRAS* G12V | 0.07 | 0.9 | 160.36 | WT | - | 6 |
| 159-MB-P (A) | III | *KRAS* G13D | 0.74 | 0.9 | 74.23 | WT | - | 5 |
| 283-MB-P (A) | III | *BRAF* V600E | 0.23 | 1.1 | 25.68 | WT | - | NA |
| 175-MB-P (A) | III | *KRAS* G12D | 0.18 | 1.3 | 9.00 | WT | - | 1 |
| 320-MB-P (A) | III | *KRAS* G12V | 0.54 | 3.0 | 127.01 | WT | - | NA |
| 197-CB-M | IV | Unknown | - | 1.1 | 7.95 | WT | - | 0 |
| 400-CB-M | IV | Unknown | - | 0.9 | 528.79 | WT | - | 1 |
| 419-CB-M | IV | Unknown | - | 0.9 | 220.99 | WT | - | 0 |
| 338-CB-M | IV | Unknown | - | 1.0 | 524.54 | WT | - | 4 |
| 382-CB-P | IV | WT | - | 1.0 | 1021.66 | WT | - | 0 |
| 390-CB-M | IV | WT | - | 0.8 | 226.79 | WT | - | 5 |
| 259-CB-P | IV | WT | - | 0.8 | 133.59 | WT | - | 0 |
| 367-CB-M | IV | WT | - | 0.8 | 1218.32 | WT | - | 3 |
| 315-CB-M-02 | IV | WT | - | 1.0 | 13.32 | WT | - | 4 |
| 418-CB-M | IV | WT | - | 0.9 | 155.82 | WT | - | NA |
| 357-CB-M | IV | WT | - | 0.9 | 106.53 | WT | - | 4 |
| 102-CB-M | IV | *BRAF* V600E | 0.66 | 1.0 | 597.25 | WT | - | NA |
| 288-CB-M | IV | *KRAS* G12D | 0.49 | 0.4 | 22.86 | WT | - | 0 |
| 277-ML-M (A) | IV | *KRAS* G12V | 0.68 | 1.2 | 837.58 | WT | - | NA |
| 321-CB-M | IV | *KRAS* G12D | NA | 0.5 | 823.27 | WT | - | 0 |
| 356-CB-M | IV | *KRAS* G12V | 0.28 | 0.9 | 282.62 | WT | - | 2 |
| 227-ML-P (A) | IV | *KRAS* G13D | 0.48 | 1.5 | 25.94 | WT | - | NA |
| 289-MW-P (A) | IV | *BRAF* V600E | 0.47 | 0.8 | 33.03 | WT | - | NA |
| 209-MB-P (A) | IV | *KRAS* G12V | 0.46 | 1.5 | 0.31 | WT | - | NA |
| **142-CB-P** | **IV** | ***KRAS* G12D** | **0.11** | **0.8** | **152.91** | ***KRAS* G12D** | **0.11** | **0** |
| **212-CB-P** | **IV** | ***BRAF* V600E** | **0.3** | **0.8** | **1015.27** | ***BRAF* V600E** | **0.01** | **NA** |
| **239-CB-P** | **IV** | ***KRAS* G13D** | **0.6** | **1.2** | **614.95** | ***KRAS* G13D** | **0.15** | **3** |
| **302-CB-M** | **IV** | ***KRAS* G12D** | **0.5** | **0.9** | **652.18** | ***KRAS* G12D** | **0.01** | **4** |
| **195-CB-P** | **IV** | ***KRAS* G12V** | **1.0** | **1.0** | **472.25** | ***KRAS* G12V** | **0.52** | **0** |
| **263-CB-M** | **IV** | ***KRAS* G13D** | **0.8** | **0.9** | **143.94** | ***KRAS* G13D** | **0.01** | **0** |
| **300-CB-P** | **IV** | ***KRAS* G13D** | **0.4** | **0.8** | **94.77** | ***KRAS* G13D** | **0.09** | **0** |
| **384-CB-M** | **IV** | ***KRAS* G13D** | **0.5** | **0.9** | **101.54** | ***KRAS* G13D** | **0.07** | **0** |
| **171-ML-M (A)** | **IV** | ***KRAS* G12D** | **0.1** | **1.2** | **40.18** | ***KRAS* G12D** | **0.05** | **2** |
| **398-CB-M** | **IV** | ***KRAS* G12D** | **NA** | **3.0** | **31.63** | ***KRAS* G12D** | **0.23** | **0** |

Patients with detectable mutations in plasma are highlighted in bold. †For patient 374-CB-M only data regarding the stage II colon cancer (G12C), but not the synchronous stage IV tumor of the pancreas (G12D) was listed. MAF: mutant allele frequency, CTCs: circulating tumor cells, WT: wild-type, NA: not available.
